# Chloroform Poisoning, Treated with Hypodermic Injections of Strychnia and Whiskey

**Published:** 1880-11-20

**Authors:** W. Perrin Nicolson

**Affiliations:** Professor of Anatomy in Southern Medical College, Atlanta, Georgia


### CASE OF CHLOROFORM POISONING, TREATED WITH HYPODERMIC INJECTIONS OF STRYCHNIA AND WHISKEY.

BY W. PERRIN NICOLSON, M.D.,

Professor of Anatomy in Southern Medical College, Atlanta, Georgia.

I was called upon the night of the 28th of September to see a woman who was reported as dying from suicidal dose of chloroform. I learned that she had taken, in the presence of a witness, about *one ounce* of chloroform.

Some time elapsed before the supervention of any toxic symptoms, so much so that those who saw her thought she was simply attempting to alarm them. In the mean time she was seen by Dr. W. G. Owen, who administered an emetic of ipecac, in order to expel any unabsorbed chloroform.

Soon after being conveyed to the police headquarters, she became profoundly narcotized, and one of the City Physicians was called to see her. He remained with her until about 8 o'clock p.m., when he left her, thinking she would die in a short time.

About 10 o'clock p.m., I was called to see her, and found the following condition. There was most profound narcosis with stertorous breathing; the cheeks and face were pallid and cold; there was entire loss of sensibility of the conjunctiva, the eyes remaining opened or closed, accordingly as they were left; pulse 80 and weak; respirations 45 per minute, and difficult on account of large collection of mucus in the throat; in fact, a typical case of profound narcotic poisoning. The thermometer in the axilla registered 94.7° F.

In order to be certain as to the temperature it was taken in the axilla three times with the same result, then in the vagina, when it registered 95.2° F.

Dr. Westmoreland had arrived a few moments before me, and was administering caffeine as an antidote. On account of the difficulty of swallowing, at my suggestion it was then given hypodermically in one-grain doses, and after each dose there was temporary improvement in the force of the pulse, but no other symptom which could be considered favorable. I then proposed that we administer strychnia and whiskey hypodermically, combined with carbonate of ammonia. On account of an unfortunate delay in getting the prescriptions it was an hour before we could begin the remedies, and by this time she had grown so much worse that I considered the case hopeless. However, at 12 o'clock we injected into the arm carb. ammon., grs. ij, in whisky ʒj, following this quickly with strychnia sulph, gr. 1–48. In about ten or fifteen minutes she moved her head from side to side, and in a short time, by coughing, succeeded in expelling the mucus which threatened to produce suffocation. She then continued to improve, soon speaking to those about the bed. In half an hour half of the above dose of whisky and ammonia, and about the same amount of strychnia was administered, after which she rested quietly until morning, except when interrupted by vomiting, which was for some hours troublesome. Large quantities of viscid mucus were ejected at times. The next morning she was much improved, and was moved to the Atlanta Hospital, when she passed from under our charge. She continued to improve steadily, and is now well.

The principal points of interest in this case seem to be : The amount taken (probably one ounce); the length of time before narcotic symptoms appeared; and the prompt action of the ammonia, strychnia and whisky.

